# Prolactin Stimulates Precursor Cells in the Adult Mouse Hippocampus

**DOI:** 10.1371/journal.pone.0044371

**Published:** 2012-09-04

**Authors:** Tara L. Walker, Jana Vukovic, Margaretha M. Koudijs, Daniel G. Blackmore, Eirinn W. Mackay, Alex M. Sykes, Rupert W. Overall, Adam S. Hamlin, Perry F. Bartlett

**Affiliations:** 1 Queensland Brain Institute, The University of Queensland, Brisbane, Australia; 2 Centre for Regenerative Therapies Dresden, Technische Universität Dresden, Dresden, Germany; Center for Regenerative Therapies Dresden, Germany

## Abstract

In the search for ways to combat degenerative neurological disorders, neurogenesis-stimulating factors are proving to be a promising area of research. In this study, we show that the hormonal factor prolactin (PRL) can activate a pool of latent precursor cells in the adult mouse hippocampus. Using an *in vitro* neurosphere assay, we found that the addition of exogenous PRL to primary adult hippocampal cells resulted in an approximate 50% increase in neurosphere number. In addition, direct infusion of PRL into the adult dentate gyrus also resulted in a significant increase in neurosphere number. Together these data indicate that exogenous PRL can increase hippocampal precursor numbers both *in vitro* and *in vivo*. Conversely, PRL null mice showed a significant reduction (approximately 80%) in the number of hippocampal-derived neurospheres. Interestingly, no deficit in precursor proliferation was observed *in vivo*, indicating that in this situation other niche factors can compensate for a loss in PRL. The PRL loss resulted in learning and memory deficits in the PRL null mice, as indicated by significant deficits in the standard behavioral tests requiring input from the hippocampus. This behavioral deficit was rescued by direct infusion of recombinant PRL into the hippocampus, indicating that a lack of PRL in the adult mouse hippocampus can be correlated with impaired learning and memory**.**

## Introduction

The existence of life-long neurogenesis in the adult mammalian brain suggests the potential for regenerative plasticity is much greater than originally expected. The subgranular zone (SGZ) of the hippocampus and the subventricular zone (SVZ) of the lateral ventricle are the two main sites of neurogenesis in the adult mammalian brain. Although SVZ neurogenesis appears important for olfaction, new neuron generation in the hippocampus is thought to be the underlying mechanism for learning and memory, novel object recognition, and spatial navigation. Neurogenesis is a multistep process, which includes activation and proliferation of neural stem cells, fate specification of precursor cells into neuronal and glial phenotypes, and synaptic integration of newborn neurons into existing neural networks [1], [2]. A variety of experience-based paradigms result in increased activation of precursors and, in turn, increased hippocampal neurogenesis [3]–[6]. Unravelling the molecular machinery of experience-induced precursor proliferation, such as diffusible factors that regulate activation of precursors, comprises one of the most active and promising areas in neural stem cell biology [7]. The identification of the factors capable of stimulating neurogenesis will have immense implications for cell replacement therapy after injury or degenerative neurological disorders.

Previous work mimicking experience-induced neurogenesis, the application of depolarizing levels of KCl to adult hippocampal cells *in vitro* revealed the presence of a latent pool of precursor cells, including stem cells, in the adult mouse brain [8]. The mechanism by which such an effect is exerted remains unclear; however, we suggest the release of various depolarization-induced factors as one possibility. Using microarray analysis, this study reveals prolactin (PRL) as one of the factors released by the adult hippocampal cells following KCl stimulation. We therefore propose PRL as a candidate for the regulation of precursor cell activation.

PRL belongs to a family of related hormones, including growth hormone and placental lactogen, and has been a major focus of research into regulation of lactation. However, in recent years, a number of reports have linked PRL to adult neurogenesis. Neurogenesis is increased in the SVZ following peripheral and central administration of PRL in both males and females or after male pheromone-induced PRL release in females [9]–[11]. In the hippocampus, PRL administration counteracts the negative effects of chronic stress exposure on neurogenesis [12]. PRL signaling is also involved in paternal offspring recognition and is associated with increased neurogenesis in the paternal olfactory bulb and hippocampus [13].

In the present study, we investigated PRL during precursor cell activation. We have demonstrated that exogenous PRL can stimulate hippocampal precursor cells both *in vitro* and *in vivo*. Conversely, PRL deficient mice have a reduced number of hippocampal precursors *in vitro* and exhibit impairments in hippocampal-dependent learning and memory tasks. These findings indicate that PRL is a promising factor for the stimulation of adult hippocampal neurogenesis.

## Materials and Methods

### Animals

C57Bl6 mice were originally obtained from the Jackson Laboratory. PRL^+/−^ [14] mice were kindly provided by Associate Professor Chris Ormandy (Garvan Institute of Medical Research, The University of New South Wales, Australia) and the colony maintained by the University of Queensland animal facility. The University of Queensland Animal Ethics Committee approved all procedures (approval numbers QBI/735/08/NHMRC and QBI/459/07). Sibling animals were genotyped as described previously [14]. All experiments were conducted in accordance with the Australian Code of Practice for the Care and Use of Animals for Scientific Purposes, with approval from the University of Queensland Animal Ethics Committee. Animals were maintained on a 12-hour light/dark cycle with food and water provided *ad libitum*.

### Tissue Dissection and Dissociation

Adult (6–8 week old) C57Bl6 mice were sacrificed by cervical dislocation, their brains immediately removed and the hippocampus or SVZ dissected. The tissue was enzymatically digested with 0.05% trypsin-EDTA (Gibco/Invitrogen) for 7 minutes at 37°C, before being washed with 0.014% w/v trypsin inhibitor (type I-S from soybean; Sigma-Aldrich) dissolved in Hepes-buffered minimum essential medium (HEM), which consisted of minimum essential medium (Gibco/Invitrogen) supplemented with 16 mM HEPES (Sigma-Aldrich) and 100 units/ml penicillin/streptomycin (Gibco/Invitrogen). Alternatively, adult hippocampal tissue was digested by incubation in a mixture containing 0.1% papain (Worthington Biochemical Corporation) and 0.1% DNaseI (Roche Australia) in Hank’s buffered salt solution (Thermo Scientific) for 20 minutes at 37°C; the mixture was triturated twice during the incubation period. Following incubation, the hippocampal and SVZ tissues were each centrifuged at 100 rcf for 5 minutes, the pellet was resuspended in 1 ml of neurosphere growth medium, mechanically triturated until smooth, and filtered through a 40 µm cell sieve (Falcon/BD Biosciences). The neurosphere growth medium consisted of mouse NeuroCult™ NSC basal medium plus mouse NeuroCult™ NSC proliferation supplements (Stem Cell Technologies) with 2% bovine serum albumin (BSA; Gibco/Invitrogen) and 2 µg/ml heparin (Sigma-Aldrich). The following growth factors were also included: 20 ng/ml purified mouse receptor-grade epidermal-like growth factor (BD Biosciences Australia) and 10 ng/ml recombinant bovine fibroblast growth factor-2 (Roche). After centrifugation at 100 rcf for 5 minutes, the cells were plated at the appropriate density in neurosphere growth medium and incubated at 37°C in a humidified 5% CO_2_ incubator for either 7 (SVZ) or 12 days (hippocampus).

### Microarray Analysis

Hippocampal tissue was dissected and dissociated, as described above, and incubated in the presence or absence of 15 mM KCl for 24 hours. Cells were centrifuged to form a pellet. Total RNA was isolated using the RNeasy mini kit (Qiagen) and quantified using the Bioanalyzer 2100 (Agilent Technologies). Antisense RNA (aRNA) was prepared from 500 ng of RNA using the Message-Amp™-II Biotin Enhanced Kit (Ambion). A total of 15 µg biotinylated aRNA was fragmented and run on a GeneChip mouse Genome 430A 2.0 Array (Affymetrix). Four microarrays were used, two for each condition. Arrays were normalized using the Affymetrix MAS5 algorithm as implemented by the *affy* package in R/Bioconductor (http://www.bioconductor.org) and a t-test filter with a threshold of p<0.05 was used. Fold changes were calculated as the difference of the means of the two arrays in each group.

### Western Blot Analysis

Tissue from the SVZ and hippocampus of C57Bl6 mice was dissected and dissociated as described above. Cells were lysed in ice-cold RIPA buffer (150 mM NaCl, 1% NP-40, 0.5% deoxycholic acid, 0.1% SDS, 50 mM Tris pH 8) supplemented with protease inhibitors (Roche). Western blots were performed using standard methods. Membranes were probed with rabbit-anti-PRL receptor antibody (Abnova) diluted 1/1000 in phosphate buffered saline (PBS) containing 5% skim milk powder (Thermo Scientific), and incubated for 90 minutes at room temperature. Membranes were then washed 6 times with PBS-T (PBS +0.05% Tween-20) over 20 minutes to remove unbound antibody, before being incubated with anti-rabbit horseradish peroxidase (1/25000; Bio-Rad Australia) for 45 minutes at room temperature. Finally, membranes were washed as described above and developed using the SuperSignal West Pico kit (Thermo Scientific).

### Primary Neurosphere Cultures

Adult hippocampal or SVZ tissue was dissected and dissociated, as described above, and the cells were plated at a density of approximately one hippocampus or SVZ per 96-well plate (Falcon/BD Biosciences) with 200 µl neurosphere medium per well. Embryonic day 14 (E14) and postnatal day 2 (P2) mice were anesthetized using ice, after which the brains were removed. The mesenchphalon and telencephalon were isolated from E14 samples; the hippocampus was dissected from P2 mice. In both cases cells were plated at a density of 500 cells per well. Cultures were supplemented, where appropriate, with either PRL or Wnt3a (recombinant mouse; R & D Systems). Primary adult hippocampal cells were incubated for 14 days at 37°C to permit neurosphere formation; adult SVZ, E14 and P2 hippocampal cells were incubated for 7 days. The primary neurospheres were then counted and sized using the 10 × objective of a bright field microscope fitted with an ocular graticule micrometer, before being collected for either passaging or differentiation. Results of the neurosphere counts were expressed as mean ± standard error of the mean (SEM).

For the conditioned medium experiments, hippocampal cells were dissociated and plated as described above. Following two days in culture, the cells were pelleted by centrifugation at 3000 rpm. The medium, conditioned in either the presence or absence of KCl, was dialyzed and concentrated using a Centriprep YM-10 column (Millipore), according to the manufacturers instructions. The concentrated medium was then diluted to its original volume in fresh NSA E+F medium and used for the culture of primary hippocampal neurospheres.

### Neurosphere Differentiation and Immunocytochemistry

Neurospheres were plated onto poly-d-lysine coated coverslips in NeuroCult™ NSC basal medium containing mouse NeuroCult™ NSC proliferation supplements without growth factors. The neurospheres were allowed to differentiate at 37°C for 5 days in humidified 5% CO_2_, at which time they were flattened and adherent. The differentiated neurospheres were then fixed with 4% paraformaldehyde (PFA; Sigma-Aldrich) in 0.1 M PBS at room temperature for 30 minutes. After washing with PBS, the neurospheres were incubated in blocking solution (5% fetal calf serum plus 5% normal goat serum (Sigma-Aldrich) in 0.1 M PBS containing 0.1% Triton X-100 (Sigma-Aldrich)) for 60 minutes at room temperature. Neurospheres were then incubated in fresh blocking solution containing mouse monoclonal βIII tubulin antibody (1∶2000; Promega) plus rabbit polyclonal glial fibrilary acidic protein (GFAP) antibody (1∶500; DakoCytomation) for 60 minutes at room temperature. The cells were washed with PBS and incubated in fresh blocking solution containing Alexa Fluor 568 anti-mouse antibody (1∶1000; Molecular Probes/Invitrogen), Alexa Fluor 488 anti-rabbit antibody (1∶1000; Molecular Probes/Invitrogen) and 4,6-diamidino-2-phenylindole (DAPI; 1∶5000; Sigma-Aldrich) for 30 minutes at room temperature. Following another PBS wash, the slides were mounted using fluorescence mounting medium (DakoCytomation) before being viewed on a Zeiss upright fluorescence microscope. A digital camera linked to Axioscope version 4 software was used for image capture.

### Hippocampal Neurosphere Passaging

Hippocampal neurosphere cultures were initiated by removing 150 µl of the medium from wells containing single neurospheres, treating with 100 µl 0.1% trypsin-EDTA for 2 minutes at room temperature, and washing with 100 µl trypsin inhibitor in HEM. The neurospheres were then mechanically triturated until dissociated and re-plated in 24-well plates in 2 ml of neurosphere medium. Neurospheres were passaged every 10 days by centrifuging the cultures, removing the medium and incubating in 1 ml of 0.1% trypsin-EDTA for 2 minutes at room temperature. After the addition of an equal volume of trypsin inhibitor, the neurospheres were centrifuged at 100 rcf for 5 minutes and the supernatant removed. Cells were mechanically triturated in 500 µl of neurosphere medium and the number of viable (trypan blue negative) cells as well as the total number of cells, were evaluated using a hemocytometer. The passaged cells were then re-plated with complete media at a density of 1×10^4^ cells/cm^2^ in tissue culture flasks (Nunc) or tissue culture plates (Falcon/BD Biosciences) as appropriate.

### Quantification of *in vivo* Neurogenesis

To quantify bromodeoxyuridine (BrdU)-positive cells, mice received a single intraperitoneal (i.p.) injection of BrdU (45 mg/kg body weight), dissolved in 0.9% NaCl (Sigma) 2 hours prior to perfusion. Animals were perfused with 0.1 M PBS followed by 4% PFA. Brains were removed and incubated overnight in 4% PFA, followed by further overnight incubations in 20% sucrose then 30% sucrose in 0.1 M PBS at 4°C. Frozen sections (50 µm) were cut using a sliding microtome. Every sixth section (approximately 7 sections per animal) was stained and mounted. For BrdU/DCX immunohistochemistry, sections were first denatured with 2 N HCl for 30 minutes at 37°C, then washed briefly in PBS. All sections were incubated for 60 minutes at room temperature with blocking solution as described above. Sections were then incubated, with rat anti-BrdU antibody (1∶100; Auspep, Melbourne, Australia) and rabbit polyclonal DCX antibody (1∶500; Abcam, Cambridge, MA), at 4°C overnight. Following washes with PBS, sections were incubated for 40 minutes at room temperature in blocking solution containing either Alexa Flour 488 donkey anti-rat secondary antibody (1∶1000), or Alexa 568 anti-rabbit antibody (1∶1000; Molecular Probes/Invitrogen), and DAPI (1∶1000). After washing with PBS, the slides were coverslipped with fluorescence mounting medium and all BrdU- or DCX-positive cells in the dentate gyrus of the hippocampus were counted using a Zeiss AxioObserver with Colibri illumination.

### Infusion of PRL into the Hippocampus

Osmotic mini pumps (Alzet, #1007D; 7 day infusion at a flow rate of 0.5 µl/hour) were loaded with PRL (recombinant mouse, R & D Systems) supplemented with 0.1% BSA or vehicle solution (0.9% sterile physiological saline, containing 0.1% BSA), attached to the infusion cannula and the entire apparatus incubated at 4°C overnight. Before surgery the osmotic mini pumps were incubated at 37°C for 2 hours. Adult C57Bl6 mice were anesthetized via intraperitoneal injection of ketamine (50 mg/kg) and xylazine (8 mg/kg), mounted onto a stereotaxic frame, and a 1.5 cm incision was made to expose the skull. A single hole was drilled in the skull (1.25 mm width) directly above the hippocampus (anterior/posterior −1.3, dorsal/lateral +1.0, dorsal/ventral −2.2, relative to Bregma), and a 30-gauge cannula was lowered and fixed to the skull using cyanoacrylate adhesive, to enable unilateral infusion directly into the hilus region of the hippocampus. Mice implanted with pumps containing PRL received 0.8 µg of PRL per day over a 7-day period. At the completion of the infusion period, mice were sacrificed, the hippocampus and SVZ dissected and neurospheres cultured. For the longer-term PRL infusion experiments, surgery was performed as described above, the only difference being that PRL was infused for 28 rather than 7 days.

### Morris Water Maze Test

Spatial memory was tested using a modified version of the Morris water maze [15]. The apparatus consisted of a circular swimming pool (100 cm diameter, 20 cm high) filled with water (25–30°C), made opaque by the addition of a non-toxic white paint. The animals were required to locate a submerged platform, hidden 1.5 cm under the water surface in a fixed location, using the spatial cues in the testing room. Mice were tested for four trials per day (60 seconds, with an inter-trial interval of approximately 20 minutes) and two different alternating start points. If the animal did not reach the platform in the given time it was then guided to the platform, where it was allowed to rest for 10 seconds before being returned to its home cage. The platform was moved to a different location after day 3 of testing, prior to day 4. The mice were tested on their ability to adapt to the changed location of the platform on days 4 and 5. The time to reach the platform (latency) was recorded using a tripod-mounted video camera, and the resultant data were analyzed using EthoVision software (Noldus Information Technology).

### Porsolt Forced Swim Test

The Porsolt forced swim test was used as a measure of depressive behavior. Animals were subjected to two trials, during which they were forced to swim in an acrylic glass cylinder filled with water from which they could not escape. The first trial lasted 10 minutes; the second trial, 24 hours later, lasted 5 minutes. The time the animal spent immobile during trial two was recorded and analyzed.

### Open Field Test

The open field test was used to measure behavioral responses such as locomotor activity, hyperactivity, anxiety and exploratory behaviors. One 10-minute trial per mouse was performed, during which the mouse was placed in a square (40×40 cm) box made of white melamine. All activity was recorded using a video camera mounted above the open field and scored using the EthoVision motion-recognition software package that detects and analyzes the movements of the mouse. The video image of the open field arena was partitioned into 12 equally sized squares: 8 border squares and 4 center zone squares. The total distance travelled, average speed and time spent in various parts of the field (for example the border areas vs. the open, middle area) were measured and analyzed.

### Y-maze

The Y-maze was employed to test spatial memory. The Y-maze was composed of two perpendicular arms connected to a runway. Both of the arms and runway were 45×5×15 cm. The perpendicular arms contained different optical cues. The maze was positioned 1 m above the ground and had a camera positioned above it. During training the mice were placed in the runway with access to only one arm for a period of 3 minutes. The training arm was alternated between each mouse. Following a 20-minute intermission back in their home cage, mice were placed back in the Y-maze for 3 minutes with access to both the training and the novel arm. The first arm entered, how many times the novel arm was entered, and the total time spent in the novel arm were analyzed.

### Contextual Fear Conditioning

The apparatus used for contextual fear conditioning consisted of a chamber (30×20×20 cm) with two clear Perspex walls, two stainless steel walls, and a removable shock grid floor consisting of 32 stainless steel rods (0.5 cm diameter, 1 cm apart). Chambers were concealed within a sound-attenuating cubicle that contained a 12-volt light, an exhaust fan and a video camera. Freezing and locomotor activity were measured using Video freeze software. The criterion for a freezing event was set at an observation interval of 2 seconds, where the mouse had to remain under the motion threshold (20 pixels, 30 frames per second) for a period of 500 milliseconds. During training, the mice were placed in the apparatus mentioned above and exposed to a training environment consisting of a grid floor wiped down with 1% acetic acid, with the light and fan on. Using Video freeze, each training session was set so that the mice received five random foot shocks of 0.8 mV over the course of 20 minutes. During this period the locomotor activity and freeze count were recorded. Twenty-four hours after the training session, the mice were placed back in the conditioning chamber, under the same conditions as the initial session. Contextual memory testing involved a 4-minute session in the conditioning chamber with no foot shocks, while locomotor activity and freeze counts were recorded.

### Statistical Analysis

Experiments were repeated at least three times, using three biological replicates, and the values expressed as mean ± SEM. Results were subjected to statistical analysis using Prism software (Graphpad) and analyzed using, where appropriate, either a Student’s t-test or one-way analysis of variance (ANOVA) followed by Tukey’s post-hoc test; significance was determined at p<0.05.

## Results

### PRL is Up-regulated in Response to Depolarization

We have previously demonstrated that the adult mouse hippocampus contains a latent stem and progenitor cell population that can be activated by neural excitation [8]. In this previous study, we showed that neural excitation, mimicked by depolarization of primary hippocampal cultures, resulted in an increase in hippocampal precursor cells as evidenced by an over 3-fold increase in neurosphere numbers. In addition, we showed that the latent hippocampal precursor population could be activated *in vivo* in response to the prolonged neural activity found in status epilepticus. However, precisely how neural activity stimulates the latent precursor population *in situ* remains unclear.

In the present study we therefore set out to determine whether the K^+^ efflux that accompanies neural activity leads to release of factors that initiate precursor activity. Consistent with this hypothesis, we found that medium that had been conditioned with KCl-depolarized primary hippocampal cells, but from which the KCl was subsequently removed, led to a 1.68-fold increase in neurosphere numbers compared to cells cultured in control conditioned medium (168.3±11.2% of control, *n* = 5 experiments). To identify factors that are released in response to depolarization, we first performed a microarray screen using RNA extracted from depolarized and control primary adult hippocampal cells. This analysis revealed significant regulation of 1028 genes following depolarization. Of these genes, 648 showed significant up-regulation in response to depolarization. The gene with the largest increase following depolarization was Wnt3a with an up-regulation of more than 14-fold (p = 0.0003). This was not unexpected however, as Wnt3a has previously been shown to be involved in the regulation of hippocampal neurogenesis [16], [17]. Another gene that was highly up-regulated following depolarization was PRL (6.4-fold increase; p = 0.021). At the outset of this study, PRL had only been shown to affect SVZ/OB neurogenesis so was chosen here for characterization of its role in the hippocampus. To confirm the presence of the PRL receptor in the adult hippocampus, we first performed western blot analysis on adult SVZ and hippocampal tissue. This analysis revealed the presence of a band of approximately 36 kDa corresponding to the expected size of the short isoform of the PRL receptor (data not shown).

### Recombinant PRL Increases the Number of Hippocampal Precursors *in vitro* and *in vivo*

**Figure 1:**
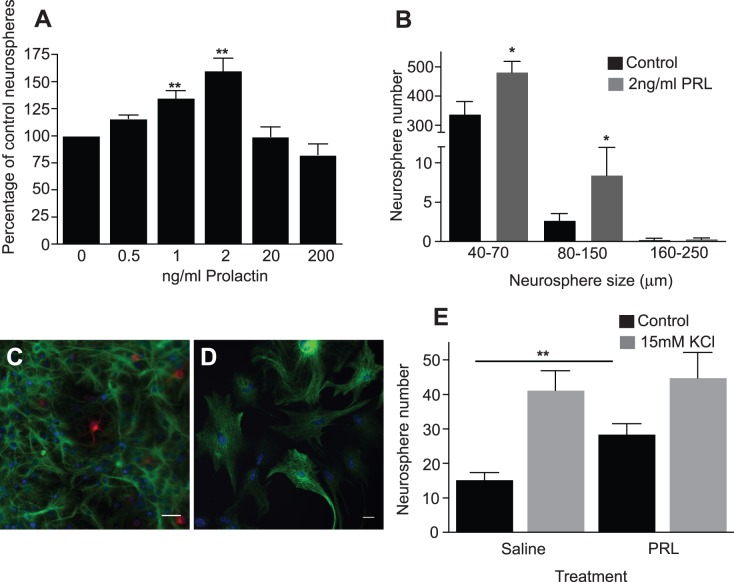
Effects of PRL on hippocampal precursors *in vitro* and *in vivo*. The addition of exogenous PRL led to a significant increase in hippocampal (***A***) derived neurospheres (*n* = 5 experiments). As well as increasing hippocampal neurosphere number, exposure of primary hippocampal cells to exogenous PRL resulted in an increase in small and medium sized neurospheres with diameters between 40 and 150 µm (***B***). Following differentiation, the large KCl-activated neurospheres contained both neurons (red) and astrocytes (green) (***C***) whereas; the smaller PRL- and control-cultured neurospheres gave rise to GFAP-positive astrocytes exclusively (***D***). Infusion of PRL directly into the adult hippocampus for 7 days resulted in a significant increase in hippocampal neurosphere numbers (***E***). Results are expressed as mean ± SEM. *p<0.05, **p<0.01. Scale bars are 20 µm.

Given the preliminary evidence that both PRL and PRLR are present in the adult hippocampus, the role of PRL in hippocampal precursor regulation was investigated. Using the *in vitro* neurosphere assay, we found a significant increase in the number of hippocampal neurospheres generated in the presence of concentrations of 1 ng/ml (135.0±6.7% of control; p<0.01; *n* = 5) and 2 ng/ml (160.1±11.4% of control; p<0.01; *n* = 5) recombinant PRL (Figure 1*A*) in the hippocampus of 2-month-old mice. Similarly, and in accordance with the results of previous studies [9], [10], the addition of 1 ng/ml (115.6±3.6% of control neurospheres; p<0.05; *n* = 5) and 2 ng/ml PRL (125.8±6.1% of control; p<0.01; *n* = 5) also led to a significant increase in the number of primary neurospheres generated from SVZ-derived cells.

PRL activation of precursors in the aged hippocampus was also tested as previous studies have demonstrated that the latent precursor population is retained in the hippocampus of older adult mice [8]. Not only did the addition of exogenous PRL significantly increase activation of a latent precursor population in 18-month-old animals, but the level of activation was in fact greater than that observed in the younger cohort (2-month-old: 160.1±11.4% of control neurospheres vs 18-month-old: 253.6±23.4% of control neurospheres; p<0.001; *n* = 4 animals per age group).

To determine whether PRL activates the more restricted progenitor and/or the stem cell population in the adult hippocampus, PRL activated neurospheres were further characterized. It is known that neurosphere size is directly related to proliferative capacity and we have previously shown that it is only very large hippocampal neurospheres, with a diameter over 250 µm, that are derived from a stem cell [8]. In the presence of 2 ng/ml PRL none of the stem cell-derived neurospheres (≥250 µm) were activated. Instead, there was an increase in the number of neurospheres in the 40–70 µm and 80–150 µm diameter range (p = 0.04; Figure 1*B*). Neurospheres generated in the presence of PRL were subjected to long-term passaging to assess their self-renewal capability. None of the neurospheres grown in PRL could be maintained long-term, in contrast to the large neurospheres previously generated in the presence of KCl [8]. In this study, we classified “long-term” as more than five passages. In addition, whereas the large KCl-activated neurospheres contained both neurons and astrocytes (Figure 1*C*), the smaller PRL- and control-cultured neurospheres gave rise to GFAP-positive astrocytes exclusively (Figure 1*D*).

Based on the above lines of evidence that demonstrate PRL-induced activation of restricted hippocampal precursors *in vitro*, the effect of exogenous PRL on adult hippocampal precursor activity *in vivo* was tested. When PRL was infused directly into the hilus of the hippocampus of 2-month-old mice for 7 days, there was an almost 2-fold increase in the number of hippocampal neurospheres generated from the PRL-infused mice compared to the saline-infused controls (28.4±3.1 vs 15.2±2.1 neurospheres per hippocampus; p<0.01; *n* = 5 animals per condition; Figure 1*E*). PRL infusion into the hippocampus also resulted in a significant increase in SVZ-derived neurospheres in the PRL-infused mice compared to the saline-infused animals (2392.4±44.1 vs 1815.8±67.2 neurospheres per SVZ; p<0.001; *n* = 5 animals per condition).

### PRL-deficient Mice Generate Less Hippocampal-derived Neuorspheres

**Figure 2:**
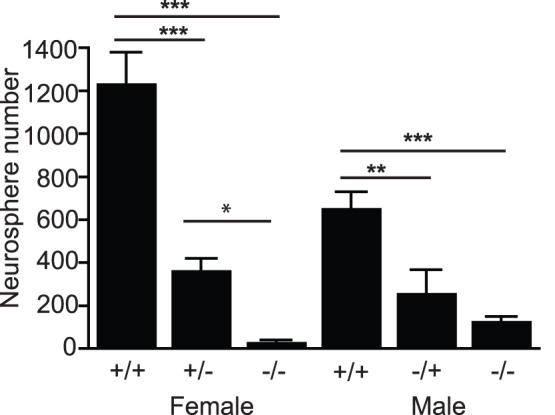
The effects of PRL-deficiency on hippocampal precursor number *in vitro*. A large decrease in hippocampal-derived neurospheres was observed in PRL^−/−^ mice compared to wild-type females (***p<0.001; *n* = 3 PRL ^+/+^ and 6 PRL^−/−^ animals) and males (***p<0.001; *n* = 9 animals each). There was also a significant decrease in hippocampal-derived neurosphere number in PRL^+/−^ compared to wild-type females (***p<0.001; *n* = 3 PRL ^+/+^ and 5 PRL^+/−^ animals) and males (***p<0.001; *n* = 6 PRL ^+/+^ and 7 PRL^+/−^ animals).

To examine whether endogenous PRL has a similar effect to exogenous PRL on adult precursor activity, the number of neurospheres derived from the hippocampus of PRL^+/+^, PRL^−/+^ and PRL^−/−^ mice was compared. A large decrease in the number of hippocampal-derived neurospheres from PRL^−/−^ compared to wild-type mice was revealed in both females (1237.5±143.5 vs 33.3±7.3 neurospheres per hippocampus; p<0.001; *n* = 3 PRL ^+/+^ and 6 PRL^−/−^ animals) and males (656±73.5 vs 130.3±19.7 neurospheres per hippocampus; p<0.001; *n* = 9 animals each; Figure 2). A significant decrease was also observed in the number of hippocampal-derived neurospheres in PRL^+/−^ mice compared to wild-type controls, again in the case of both females (1237.5±143.5 vs 366.6±53.8 neurospheres per hippocampus; p<0.001; *n* = 3 PRL ^+/+^ and 5 PRL^+/−^ animals) and males (656±73.5 vs 261.4±106.2 neurospheres per hippocampus; p<0.001; *n* = 6 PRL ^+/+^ and 7 PRL^+/−^ animals; Figure 2). No differences in body weight, brain weight or total number of hippocampal cells in the PRL null mice were observed when compared to their wild-type littermates (data not shown). The deficit in precursor numbers in the PRL^−/−^ SVZ was not significant in females (581.5±38.7 vs 425.3±121.9 SVZ-derived neurospheres per brain, *n* = 5 PRL^+/+^ and 6 PRL^−/−^ animals). Conversely, in the male mice, a significant decrease in SVZ precursors was observed (623.6±82.8 vs 405.2±83.6 SVZ-derived neurospheres; p<0.001; *n* = 9 PRL^+/+^ and 10 PRL^−/−^ animals); however, this reduction was smaller than that found in the hippocampus.

We also investigated whether this deficit was present at embryonic and early postnatal stages. At E14 no significant difference in the number of hippocampal neurospheres generated from PRL^−/−^ (65.7±5.6 neurospheres per 1000 cells), PRL^−/+^ (65.0±7.1 neurospheres per 1000 cells) and PRL^+/+^ animals was observed (66.2±8.0 neurospheres per 1000 cells). Similarly, at P2 there was no difference in neurosphere number between PRL^−/−^ (263.0±35.6 neurospheres per 500 cells), PRL^−/+^ (324.7±58.1 neurospheres per 500 cells) and PRL^+/+^ animals (259.0±37.4 neurospheres per 500 cells). For all genotypes, the addition of 2 ng/mL PRL to either P2 or E14 cultures had no effect on neurosphere number.

**Figure 3:**
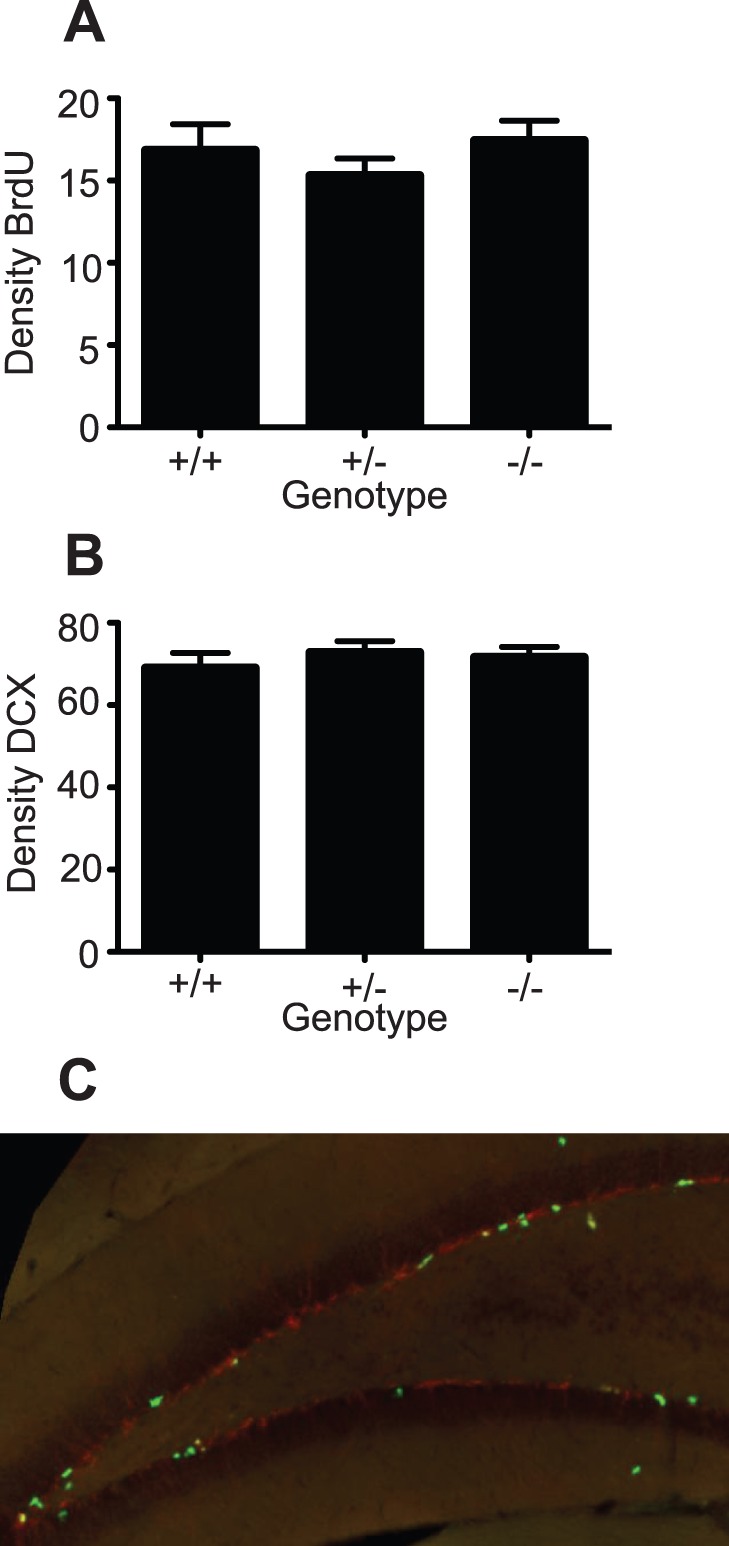
The effects of PRL-deficiency on hippocampal precursor number *in vivo*. (***A***) No difference was observed in proliferation (BrdU-positive cells; ***A***), nor in the generation of new neurons (DCX-positive cells; ***B***) in the hippocampus of PRL^−/−^ mice compared to wild-type littermates (*n* = 6 animals per genotype). (***C***) A representative image of BrdU-positive (green) and DCX-positive staining in the PRL^−/−^ hippocampus.

We next examined whether the decrease in neurosphere formation that we observed in the PRL null mice using the *in vitro* neurosphere assay would also result in a decrease in hippocampal neurogenesis *in vivo*. To address this, we stained hippocampal sections taken from PRL^+/+^, PRL^−/+^ and PRL^−/−^ animals, which had received BrdU injections, with markers for proliferating cells (BrdU) and newly born neurons (DCX). Interestingly, we found no difference in proliferation (PRL^−/−^17.44±1.2 vs PRL^+/+^16.8±1.6 BrdU-positive cells per section; Figure 3*A*) or the generation of new neurons (PRL^−/−^71.6±2.6 vs PRL^+/+^69.0±3.7 DCX-positive cells per section; Figure 3*B*) between genotypes. Figure 3*C* shows a representative image of BrdU-positive (green) and DCX-positive staining in the PRL^−/−^ hippocampus.

### The Neurosphere Formation Deficit in PRL-deficient Mice cannot be Rescued *in vitro*

**Figure 4:**
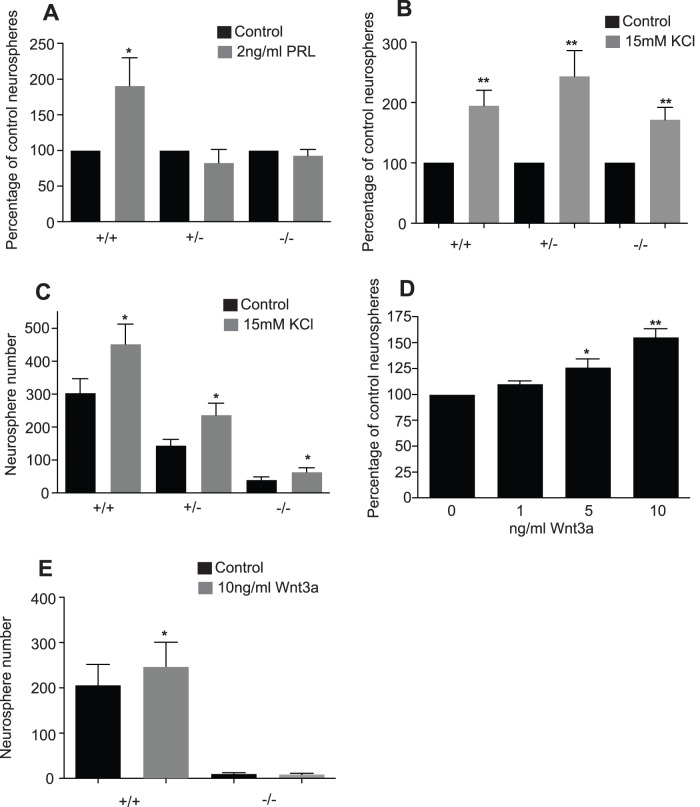
The precursor deficit in PRL-deficient mice cannot be rescued by the addition of PRL or KCl-induced depolarizarion. (***A***) The addition of 2 ng/ml PRL to cultures of hippocampal cells from adult PRL^+/+^ mice resulted in a significant increase in neurosphere numbers (*p<0.05; *n* = 6 animals). However, the addition of exogenous PRL to cultures derived from PRL^−/−^ or PRL^−/+^ hippocampi resulted in no increase in neurosphere number (not significant; *n* ≥ 6). (***B***) The PRL^−/−^, PRL^+/−^ and PRL^+/+^ hippocampus could all be activated by KCl-induced depolarization *in vitro* to generate approximately twice as many neurospheres (p<0.01; *n* = 11 animals per group). (***C***) This activation,while significant in the PRL−/− hippocampus was not enough to bring the neurosphere number back to those generated from a PRL wild-type hippocampus. (***D***) The addition of purified Wnt3a to primary hippocampal cells from wild-type mice resulted in a significant increase in neurosphere number at concentrations of 5 ng/ml and 10 ng/ml Wnt3a, with the most significant increase observed at 10 ng/ml (*p<0.05, **p<0.01; *n* = 3 experiments). (***E***) The PRL^−/−^ hippocampus produced a lower number of neurospheres and the addition of Wnt3a to these cells was unable to rescue this deficit (*p<0.05; *n* = 4 PRL^+/+^ and 5 PRL^−/−^ animals).

To test whether the deficit in hippocampal neurosphere activity in the PRL-deficient mice could be rescued, exogenous PRL was added to primary hippocampal cells from the PRL^−/−^, PRL^−/+^ and PRL^+/+^ mice. As expected, the addition of 2 ng/ml PRL to cultures of hippocampal cells from the PRL^+/+^ mice resulted in a significant increase in neurosphere numbers (190.3±36.8% of control; p<0.05; *n* = 6; Figure 4*A*). However, the addition of exogenous PRL to cultures derived from PRL^−/−^ or PRL^−/+^ hippocampi produced no significant change in neurosphere number (PRL^−/−^92.8±7.6% of control; PRL^−/+^82.5±18.6% of control; *n* = ≥6; Figure 4*A*).

The activation of latent precursors in the PRL^−/−^ hippocampus by *in vitro* depolarization was also tested. Hippocampal cells from both the PRL^−/−^ and the PRL^+/+^ hippocampus could be activated by KCl-induced depolarization (PRL^−/−^171.3±20.71% of unstimulated; PRL^+/+^194.7±25.6% of unstimulated neurospheres per hippocampus, *n* = 11; Figure 4*B*). Although the precursor cells isolated from the PRL^−/−^ hippocampus could be activated, this activation only partially rescued the deficit in precursor activity observed in the PRL^−/−^ hippocampus (PRL^−/−^39.7±9.6 vs 63.3±11.1 neurospheres per hippocampus; PRL^+/+^304.1±40.8 vs 452.4±57.4 neurospheres per hippocampus, *n* = 11; Figure 4*C*). Importantly, KCl-induced depolarization of the PRL^−/−^ hippocampal cells resulted in the generation of a number of large stem-cell derived neurospheres with the capacity to generate neurons when differentiated.

In addition to PRL and KCl, Wnt 3a, another exogenous factor that we found to be up-regulated following depolarization, was tested for its ability to activate latent precursor cells in the PRL^−/−^ mice. Wnt3a is known to increase hippocampal neurogenesis either by activity-dependent synaptic release from adult hippocampal neurons [16] or through its secretion from hippocampal astrocytes [17]. The addition of 5 ng/ml and 10 ng/ml purified Wnt3a to primary hippocampal cells from wild-type mice resulted in a significant increase in neurosphere number, with the most significant increase observed at 10 ng/ml (155.4±7.9% of control; p<0.01; *n* = 3; Figure 4*D*). Based on this finding, 10 ng/ml Wnt3a was added to primary hippocampal cells from both PRL^+/+^ and PRL^−/−^ mice and, as expected, an increase in neurosphere number was observed (Figure 4*E*; 205.5±46.3 vs 276.5±64.7 neurospheres per hippocampus; *n* = 4). The PRL^−/−^ hippocampus produced a significantly lower number of neurospheres compared to the wild-type hippocampus, and the addition of Wnt3a to these cells could not rescue this deficit (9.6±2.8 vs 8.6±2.6 neurospheres per hippocampus; *n* = 5; Figure 4*E*).

### PRL-deficient Mice have Specific Deficits in Hippocampal-dependent Learning and Memory

**Figure 5:**
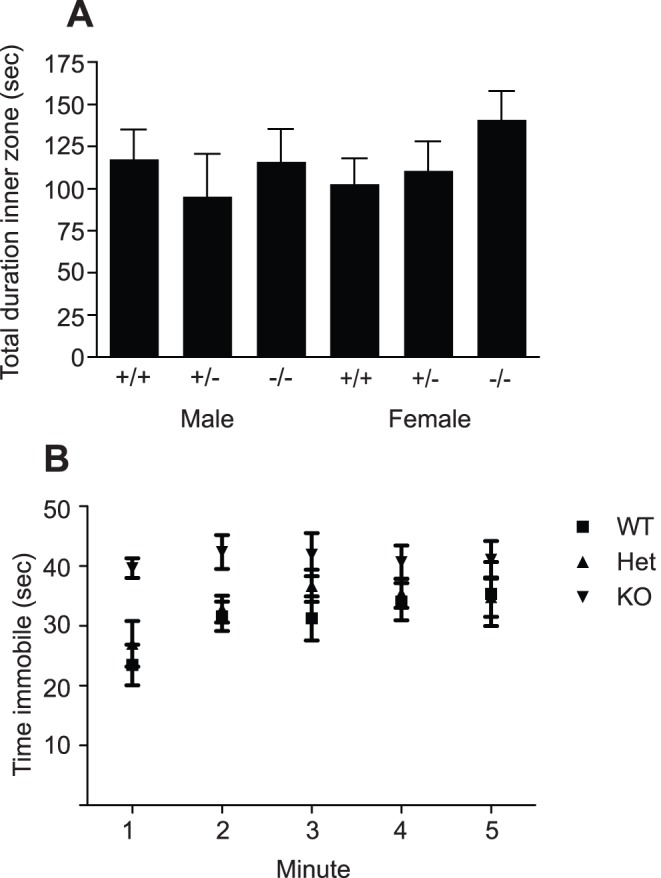
The effects of PRL deficiency on exploratory and anxiety behaviors. (***A***) In the open field test no significant differences were observed in the frequency, total duration, or latency of first occurrence in the inner zone nor in the total average distance moved between the genotypes (*n* = 43 animals). (***B***) Mice were also tested in the Porsolt forced swim test. The time that the mice spent immobile on the second day was measured and no significant difference between the PRL^−/−^, PRL^−/+^ and PRL^+/+^ mice was observed (*n* = 43).

To assess whether the deficit in hippocampal precursor number observed in the PRL-deficient mice resulted in any behavioral deficits, male and female PRL^−/−^, PRL^+/−^ and PRL^+/+^ mice were tested via a number of hippocampal-dependent and -independent tasks. In the open field test, which was designed to measure locomotor activity, hyperactivity, anxiety and exploratory behavior, no significant differences between sexes or genotypes were observed. Specifically, no change in the total average distance travelled, time spent in the inner zone, or speed was observed (Figure 5*A*). In the Porsolt forced swim test, used to assess depressive behaviors, the time that the mice spent immobile on the second day of testing was measured, with no significant difference between the PRL^−/−^, PRL^−/+^ and PRL^+/+^ mice of either sex observed (Figure 5*B*).

**Figure 6:**
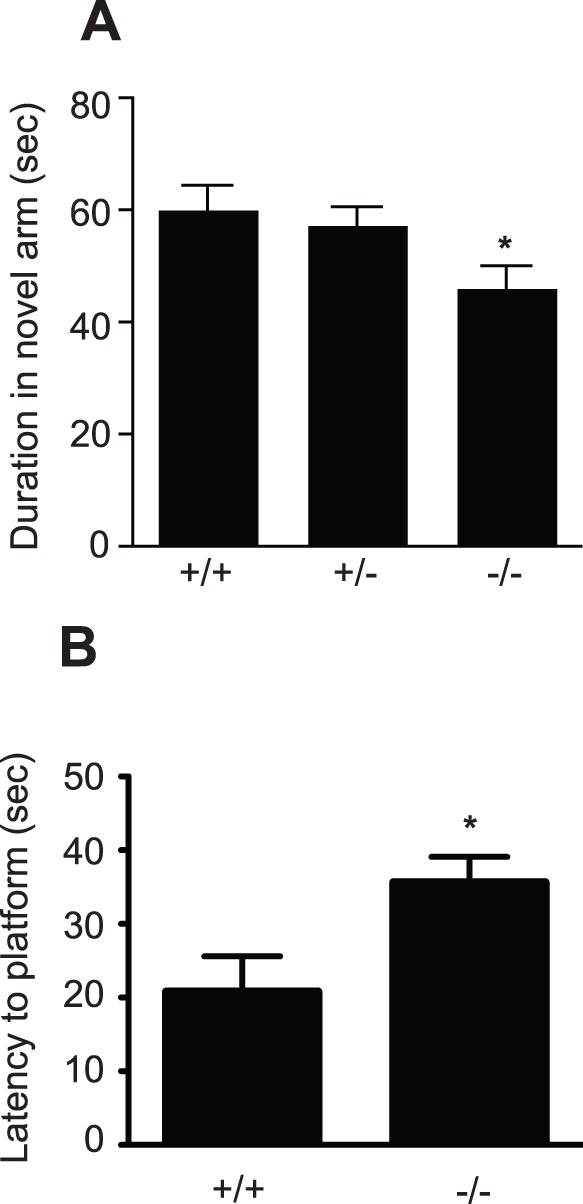
PRL deficiency results in hippocampal learning defects. (***A***) The PRL null mice spent significantly less time in the novel arm of a Y-maze compared to their wild-type littermates (*p<0.05; PRL^−/−^ *n* = 8 animals and PRL^+/+^ *n* = 9 animals). (***B***) In the reversal version of the water maze, male and female PRL^−/−^ and PRL^+/+^ mice were trained over a period of three days to locate the hidden platform. Prior to the fourth day of training the hidden platform was moved to a novel location and the mice were assessed on their ability to adapt and learn the new platform location. On day 4 the PRL^−/−^ mice took significantly longer to adapt to the novel location than the PRL^+/+^ mice (Student’s t-test *p<0.05; *n* = 6 PRL^+/+^ and 7 PRL^−/−^ animals).

Given that no deficits were observed in non-hippocampal-dependent behaviors, the two-trial recognition version of the Y-maze was then used as a test of hippocampal memory function. Mice were placed in the Y-maze, with one arm of the maze blocked, and allowed to explore for 3 minutes. Following a 20-minute inter-trial interval, the animals were placed back into the maze with both arms open and the amount of time spent in the novel arm was recorded. No difference was observed between male and female mice; however, on genotype-only comparison, i.e. male and female data combined, the PRL deficient mice spent significantly less time in the novel arm than their wild-type littermates (PRL^−/−^46±4.1 seconds; *n* = 8 vs. PRL^+/+^60.1±4.3 seconds; p<0.05; *n* = 9; Figure 6*A*), indicating impairment in hippocampal learning.

To further address the issue of hippocampal cognitive impairment, PRL-deficient mice were tested using a modified version of the Morris water maze. We used a reversal protocol of the classical water maze task [18], whereby the hidden platform was moved to a new location after three days but no spatial cues were changed. In this task robust functional plasticity of the encoding network in the dentate gyrus is required because the original location of the platform has to be omitted in favor of a new location. As expected, on day 4 the PRL^−/−^ mice took longer to adapt to the novel location than the PRL^+/+^ mice (PRL^+/+^20.79±4.8 vs PRL^−/−^35.61±3.5 seconds; p = 0.027; *n* = 7 PRL^−/−^ and 6 PRL^+/+^ animals; Figure 6*B*), further confirming the hippocampal deficit in spatial learning and memory in the PRL-deficient mice. The swim speed did not differ between the groups (data not shown).

**Figure 7:**
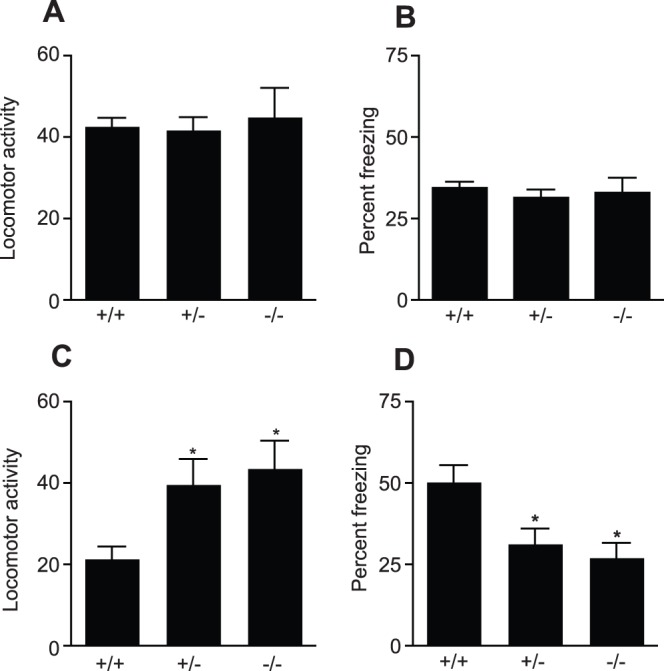
Effects of PRL deficiency on contextual fear learning and memory. During the training session no difference was observed in either the locomotor activity (***A***) or freezing (***B***) between the genotypes. Twenty-four hours after the training session, the mice were placed back in the conditioning chambers, using the same training chamber context. The PRL^−/−^ and PRL^−/+^ mice showed significantly more locomotor activity (***C***) with significantly less freezing (***D***) than the PRL^+/+^ mice (*p<0.05; PRL^+/+^ *n* = 9; PRL^−/+^ *n* = 11; PRL^−/−^ *n* = 8).

Although fear conditioning is generally thought to depend on the amygdala, some forms of this response, for example contextual and trace fear conditioning, also involve the hippocampus. PRL^−/−^, PRL^−/+^ and PRL^+/+^ mice were exposed to a training environment in which they received five random foot shocks of 0.8 mV over the course of 20 minutes. During the 20-minute session, both the locomotor activity and the total time frozen were recorded and at this point, no difference in either behavior was observed between the genotypes (Figure 7*A,B*). Twenty-four hours after the training session, the mice were placed back in the conditioning chambers using the same chamber context. Contextual memory testing involved a 4-minute session in the conditioning chamber with no foot shocks, during which locomotor activity and percentage of time spent frozen were recorded. Interestingly, the PRL^−/−^ and PRL^−/+^ mice showed significantly more locomotor activity (PRL^+/+^21.3±3.1, *n* = 9; PRL^−/+^39.7±6.2, *n* = 11; PRL^−/−^43.5±6.9, *n* = 8; Figure 7*C*) and significantly less freezing than the PRL^+/+^ mice (PRL^+/+^50.3±5.2% of total time, *n* = 9; PRL^−/+^31.3±4.8% of total time, *n* = 11; PRL^−/−^27.0±4.7% of total time, *n* = 8; Figure 7*D*), indicating an impaired ability of the PRL-deficient mice to remember the aversive context.

### Hippocampal-dependent Learning Deficits in PRL-deficient Mice can be Rescued by PRL Infusion

**Figure 8:**
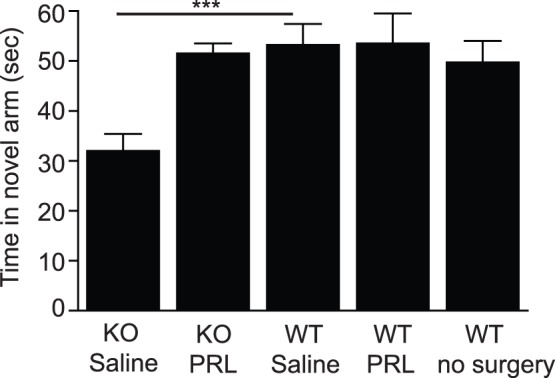
The hippocampal-dependent learning deficit in PRL null mice can be rescued by infusion of PRL into the hippocampus. Following a 28-day infusion of either PRL or saline into the hippocampus, PRL^−/−^ and PRL^+/+^ mice were tested on hippocampal-dependent learning using the Y-maze. When saline was infused into the hippocampus, the PRL null mice spent significantly less time in the novel arm compared to their wild-type littermates (***p<0.001; PRL^−/−^ *n* = 5, PRL^+/+^ *n* = 5), indicating an impairment in hippocampal learning. Following infusion of PRL into the hippocampus, PRL null mice behaved similarly to their wild-type counterparts (no significant difference; PRL^−/−^ *n* = 5, PRL^+/+^ *n* = 4), indicating that a lack of PRL in the null mice is responsible for the memory deficit.

To determine whether the hippocampal-dependent learning deficits in the PRL^−/−^ mice were due to lack of PRL in the hippocampus, either PRL or saline was directly infused into the hippocampus of PRL^−/−^ and wild-type mice over a period of 4 weeks. A cohort of wild-type mice that received no surgery was also included as a control to account for any effects of surgery on performance in the Y-maze. When saline was infused into the hippocampus of both PRL^−/−^ and wild-type mice, it was found that the PRL-deficient mice spent significantly less time in the novel arm compared to their wild-type littermates (Figure 8; PRL^−/−^32.0±3.4 seconds; *n* = 5 vs PRL^+/+^53.2±4.2 seconds; p<0.001; *n* = 5), indicating an impairment in hippocampal learning. However, infusion of PRL into the hippocampus of PRL^−/−^ mice was able to overcome this deficit, restoring behavior to levels similar to that of the wild-type controls (Figure 8; PRL^−/−^51.6±1.9 seconds; *n* = 5 vs PRL^+/+^53.5±6.0 seconds; *n* = 4). No difference between operated and non-operated wild-type mice was observed.

## Discussion

It is known that adult neurogenesis is reduced with age and in many conditions associated with neurodegeneration. To date, however, no therapeutic approach has been developed capable of modulating or reversing this decline. There is overwhelming evidence suggesting adult neural stem cells as an as yet unrealized source of new neurons to repopulate the degenerating brain. The discovery of a molecular pathway, which could either prevent the decline in neurogenesis or increase recruitment from the neural stem cell pool, would have far-reaching implications in the treatment of neurodegenerative and age-related cognitive disease.

In our previous research, we described a latent population of neural precursors in the adult hippocampus that can be triggered to generate new neurons [8]. We showed that depolarization of adult mouse hippocampal cells *in vitro,* using high levels of KCl, led to an over 3-fold increase in the number of neurospheres generated, a small number of which showed the defining stem cell properties of proliferation, self-renewal and multipotentiality. Here, we show that that depolarization of hippocampal cells leads to the release of as yet unidentified factors. Medium conditioned with KCl-depolarized primary hippocampal cells, but from which the KCl is subsequently removed, can activate precursor activity as effectively as direct application of KCl. Therefore, the aim of the current research was to characterize factors underpinning this activation, particularly molecules that can cross the blood-brain barrier to enhance brain repair in a non-invasive manner. To identify this unknown factor(s), we performed microarray analysis comparing primary hippocampal cells cultured for 24 hours in either control or depolarizing levels of KCl. These microarrays revealed a significant up-regulation of 648 genes following *in vitro* depolarization. We selected two of these candidates, Wnt3a and prolactin (PRL), for further characterization. Wnt3a has previously been shown to be involved in hippocampal neurogenesis so was chosen as a good candidate to verify the pro-neurogenic transcriptional effects of depolarization. The PRL gene, significantly up-regulated over 6-fold in response to depolarization, encodes a pregnancy hormone that is involved in regulating proliferation in many types of cells in the brain, including SVZ precursor cells [9], [19], astrocytes [20], neuroblastoma cells [21], oligodendrocyte precursor cells [22] and hippocampal precursor cells [12], [13]. PRL was chosen for further characterization, as a number of reports had already linked PRL to adult SVZ neurogenesis. The first report came in 2003 from Shingo and colleagues, who reported that pregnancy increased SVZ neurogenesis, and this effect was mediated by PRL. The same group also demonstrated that pheromones of dominant males induce a PRL-mediated increase in SVZ neurogenesis in female mice [10].

Despite initial reports claiming that PRL is not involved in hippocampal neurogenesis [10], it has been recently demonstrated that PRL counteracts the negative effects of chronic stress exposure on neurogenesis in the dentate gyrus [12] and is involved in paternal recognition [13]. These studies, however, disagree on the direct effect of PRL on hippocampal neurogenesis *in vivo* [12], [13]. The study by Mak and Weiss demonstrated that infusion of PRL into the lateral ventricle of male mice significantly increased proliferation in the dentate gyrus [13]. In contrast, the study by Torner and colleagues observed no effect of direct PRL administration on hippocampal precursor proliferation [12]; possibly due to different delivery methodology (subcutaneous injections rather than intracerebroventricular infusion) or dosage and timing of PRL administration. Also, and in contrast to our findings, an *in vitro* follow-up study published by the same group demonstrated that PRL doesn’t affect cell proliferation, differentiation or survival of NPCs using the neurosphere assay [23]. This led to their conclusion that the effect of PRL observed *in vivo* is probably not due to a direct effect of PRL on NPCs, but rather via an indirect mechanism such as PRL-induced attenuation of the HPA axis response [23]. In that study, the concentration of PRL used *in vitro* was between 10 and 1000 ng/mL, which is between 10 and 1000 times that used in the present study.

We have shown above that exogenous PRL can significantly increase the number of neurospheres generated from both the adult (2-month-old) and aged (18-month-old) hippocampus. Interestingly, exogenous PRL stimulates the more restricted progenitor cell population and not the multipotential stem cells, corroborating a recent report showing that the PRLR co-localizes with doublecortin-positive progenitor cells, rather than with stem cell markers Sox2 or GFAP, in the adult hippocampus [13]. However, this does not mean that these cells are not capable of producing neurons *in vivo* but rather indicates that they require other factors *in vitro* such as BDNF before they can form neurons [24]. In addition to the action of exogenous PRL, we have demonstrated that PRL deficiency leads to a reduced neurosphere forming activity in the adult SVZ and hippocampus *in vitro*. In contrast to the neurosphere data, *in vivo* analysis revealed no change in BrdU- or DCX-positive cells in the PRL^−/−^ hippocampus compared to that of wild-type littermates. This suggests that the precursor cells in the PRL null mice can be maintained by other niche factors secreted *in vivo*, unlike in the *in vitro* situation where the cells are isolated from the milieu of *in vivo* niche factors and the PRL deficit alone is sufficient to significantly decrease their proliferation. Alternatively, it is possible that PRL is affecting either the stem cell number, mediating the cell cycle dynamics of the proliferating precursor cells, or affecting new neuron maturation or survival. The BrdU paradigm used in this study (an acute snapshot of cells dividing during a 2 hour time window) is unlikely to label many of the “Type-1” stem cells as these cells are slowly and infrequently dividing and this paradigm will mostly label the fast dividing transit amplifying cells. Even if some of the stem cells are labeled this will only constitute an extremely small proportion of the total BrdU-labeled proliferating cells masking any possible changes in stem cell number. Unfortunately good antibodies for the immunohistochemical differentiation between the Type 1 stem cells and the faster dividing Type 2 precursors are not available and most of the commonly used “stem cell” markers such as Sox2 and Nestin label both populations. *In vitro* using the neurosphere assay in the presence of high levels of EGF/FGF the cells (even the slower dividing stem cells) are stimulated to proliferate rapidly. Therefore, it is possible that the PRL KO hippocampus contains less stem cells explaining why we can detect a deficit using the neurosphere assay but not by acute BrdU labeling. It is also possible that PRL has an effect on net neurogenesis (survival) and this could be determined by a BrdU pulse chase experiment whereby proliferating cells are labeled with BrdU and after four weeks the number of new neurons (BrdU/NeuN double positive cells) are evaluated. It is also possible that PRL could affect the newborn cell maturation speed without directly changing either the proliferation or the net survival. While these may be interesting information, elucidating the complex effects of PRL on cell cycle dynamics and maturation are beyond the scope of this study.

Given the reduced precursor activity in these mice, it was important to determine whether the precursor population was largely absent or whether precursors were latent and might be activated by the addition of exogenous PRL. In fact, we discovered that the addition of PRL had no effect in the PRL^−/+^ and PRL^−/−^ cultures. It is likely, however, that PRL^−/−^ hippocampal precursors cannot respond to exogenous PRL due to the short PRL exposure and/or possible PRLR down-regulation. This prompted us to investigate the effects of KCl depolarization, as our previous studies showed that the latent precursor cell population could be activated by the factor/s released following depolarization. The latent precursor cell population in the PRL null mice could be activated to some extent by KCl depolarization, but not to levels observed in the unstimulated PRL^+/+^ hippocampus. This indicates that while PRL null mice do possess a latent hippocampal precursor population capable of being activated by depolarization, this recruitable population is smaller than in wild-type mice.

The constant turnover of neurons in the adult hippocampus is required for the processing of new spatial memories [18]. In line with the deficit in hippocampal precursor numbers in the PRL null mice, we observed accompanying hippocampal-based behavioral deficiencies in these animals. Significant deficits in the three behavioral tests requiring input from the hippocampus were observed, with no apparent deficits recorded in non-hippocampal tests. Crucially, we demonstrated that the deficit in hippocampal learning observed in the PRL null mice was due to lack of PRL, as infusion of exogenous PRL into the PRL^−/−^ hippocampus for a period of 4 weeks restored the deficit observed in the Y-maze to wild-type levels. It is possible that the improvement in behavior following infusion of PRL in to the PRL null mice could be due to other PRL-associated changes not directly affecting neurogenesis. Since neurogenesis resulting from PRL stimulation has been shown to influence behaviors such as paternal recognition and care [13], however, it is reasonable to suggest that the ability to restore the observed deficit occurs via this process. Mak and colleagues found that mice deficient in PRLR, either induced via intracerebroventricular infusion of a PRL neutralizing antibody or by the use of PRLR^−/−^ animals, did not exhibit the enhanced cell proliferation observed following paternal interaction in the dentate gyrus of PRL^+/+^ mice [13]. In addition, this deficit could be rescued by enhancing hippocampal neurogenesis in PRLR null animals using luteinizing hormone.

The action of PRL is mediated through its binding to PRLR. PRLR^−/−^ mice exhibit a marked deficiency in maternal care [25]. However, using the Morris water maze, these mice have been shown to acquire spatial learning at the same rate as PRLR wild-type animals, and to perform similarly in the probe test [25]. Based on these findings, the authors postulated that, because maternal behavior is regulated by the hypothalamus, the defect in PRLR mutants could be limited to the hypothalamus. Interestingly, we observed no deficit in precursor numbers in either the SVZ or hippocampus of PRLR null mice (data not shown). This is in agreement with previous work by Mak and colleagues, who also showed no difference in BrdU-labeled cells in the dentate gyrus of PRLR^−/−^ and PRLR^+/+^ mice under baseline conditions [10]. It was only following exposure to male pheromones that an increase in proliferation, compared to baseline, was observed in either the PRLR^−/−^ or PRLR^+/+^ females.

Unraveling the molecular machinery of experience-induced precursor proliferation, such as diffusible factors that regulate activation of precursors, comprises one of the most active and promising areas in neural stem cell biology [7]. in this study, we have identified PRL as one such factor that may play an important role in activating the endogenous hippocampal precursors in cases of neurodegenerative disease.
